# Phylogenetic and structural insights into the origin of C-type lectin Mincle in vertebrates

**DOI:** 10.1007/s00251-025-01375-x

**Published:** 2025-03-22

**Authors:** Taiki Ito, Carla Guenther, Eri Ishikawa, Takae Yabuki, Masamichi Nagae, Yoichiro Nakatani, Sho Yamasaki

**Affiliations:** 1 Department of Molecular Immunology, Research Institute for Microbial Diseases, Osaka University, Suita, Osaka Japan; 2 Laboratory of Molecular Immunology, Immunology Frontier Research Center (IFReC), Osaka University, Suita, Osaka Japan; 3 Center for Advanced Modalities and Drug Delivery Systems (CAMaD), Osaka University, Suita, Osaka Japan; 4 Laboratory of Medical and Evolutionary Genomics, Department of Biological Informatics, Bioinformatics Center, Research Institute for Microbial Diseases, Osaka University, Suita, Osaka Japan; 5 Center for Infectious Disease Education and Research (CiDER), Osaka University, Suita, Osaka Japan

**Keywords:** C-type lectin receptors, Molecular phylogenetics, Crystal structure, Ligand specificity, FcRγ, Vertebrates

## Abstract

Our bodies are continuously exposed to injurious insults by infection and tissue damage, which are primarily sensed by innate immune receptors to maintain homeostasis. Among such receptors is macrophage-inducible C-type lectin (Mincle, gene symbol *CLEC4E*), a member of the C-type lectin receptor (CLR) family, which functions as an immune sensor for both pathogens and damaged self. To monitor these injurious stimuli, Mincle recognizes disaccharide-based pathogen-derived glycolipids and monosaccharide-based intracellular metabolites, such as β-glucosylceramide. Mincle is well-conserved among mammals; however, there are questions that remain unclear, such as from which lower vertebrate did it arise and whether the original ligand was self or non-self. Here, we found homologues of Mincle and its signaling subunit Fc receptor γ chain (FcRγ) in lower vertebrates, such as reptiles, amphibians, and fishes. The crystal structure of a Mincle homologue revealed that fish Mincle possesses a narrower sugar-binding pocket than that of mammalian Mincle, and accommodates only monosaccharide moieties. These results suggest that Mincle may have evolved from a self-recognizing receptor, and its sugar-binding pocket widened during evolution, presumably to adapt to disaccharide-based glycolipids derived from life-threatening pathogens.

**Supplementary Information:**

The online version contains supplementary material available at 10.1007/s00251-025-01375-x.

## Introduction

Many multicellular organisms are primarily protected from environmental pathogens by establishing the innate immune system. Innate immune sensors recognize and eliminate non-self-pathogens, while they are generally considered to be tolerant to self. Recently, however, it has been demonstrated that innate immune sensors also bind to self-molecules (nucleic acids, lipids, carbohydrates, proteins, metabolites, etc.), presumably to detect internal and external perturbation on the body by sensing quantitative/qualitative alteration of self-components (Gong et al. 2020). C-type lectin receptors (CLRs) are well-conserved innate immune sensors in mammals that recognize glycoconjugates derived from self and non-self and induce innate immune responses (Reis et al. 2024).

Macrophage-inducible C-type lectin (Mincle) is a CLR expressed by innate immune cells, like macrophages and dendritic cells. Upon ligand recognition, Mincle transduces downstream signals via Fc receptor γ chain (FcRγ), an immunoreceptor tyrosine-based activation motif (ITAM)-containing adaptor protein (Yamasaki et al. 2008). Mincle recognizes its ligands via a calcium-dependent sugar-binding site, containing a conserved EPN (Glu-Pro-Asn) motif, and a hydrophobic groove on the carbohydrate recognition domain (CRD), which are in combination the major characteristics of Mincle. Using these structural characteristics, Mincle senses dangers and activates immune responses by recognizing a variety of pathogen-derived glycolipids one of them being trehalose dimycolate (TDM) (Ishikawa et al. 2009) or damaged self-derived β-glucosylceramide (β-GlcCer) (Nagata et al. 2017). However, whether Mincle originated as a receptor for pathogens or self-ligands remains unclear.

In this study, we searched for and compared Mincle homologues present in reptiles, amphibians, and fishes. Structural analysis revealed that fish Mincle has a narrower sugar-binding pocket than that of mammalian Mincle, suggesting that Mincle was originally a receptor for self-derived monosaccharide-bearing glycolipids, and especially between amphibian and reptile, evolved to recognize disaccharide-bearing glycolipids, presumably derived from pathogens.

## Materials and methods

### Sequence analysis via BLAST search

Gene, cDNA, and protein sequences were obtained from the National Center for Biotechnology Information (NCBI) database. The longest isoform of human Mincle (huMincle) and FcRγ (huFcRγ) was selected and used for further analysis and experiments. Protein BLAST (NCBI) analysis was performed for huMincle and huFcRγ homologues in non-mammalian vertebrates. Specifically, the vertebrates (taxonomy ID: 7742) were selected as a search set while excluding mammals (taxonomy ID: 40674). Also, BLAST search for huMincle and huFcRγ homologues was conducted in the following individual species: *Polyodon spathula* (Mississippi paddlefish, taxonomy ID: 7913), *Synaphobranchus kaupii* (Kaup’s arrowtooth eel, taxonomy ID: 118154), and *Takifugu rubripes* (Japanese pufferfish, taxonomy ID: 31033), respectively. A BLAST search was conducted using the full-length amino acid sequence of huMincle as a query against the *T. rubripes* genome. Among the top 10 hits, the full-length amino acid sequences were aligned with huMincle, and the highest-ranking molecule that retained the EPN motif and the hydrophobic amino acids forming the hydrophobic groove was designated as trMincle. Lastly, for searching huMincle homologues in reptiles and amphibians, we selected reptiles (taxonomy ID: 8459) and amphibians (taxonomy ID: 8292) as a search set.

### Cells

HEK293 (Thermo Fisher Scientific) cells were cultured with Dulbecco’s modified eagle medium supplemented with 10% (v/v) fetal bovine serum and penicillin/streptomycin in 37 °C at 5% CO_2_.

### Transfection and expression check of Takifugu rubripes Mincle

The DNA fragments encoding FLAG-tagged human FcRγ (huFcRγ) and *Takifugu rubripes* FcRγ (trFcRγ) and HA-tagged huMincle and *Takifugu rubripes* Mincle (trMincle) were separately cloned into pcDNA3.1 vector. The constructed plasmids were then transfected into HEK293 cells using PEI MAX (Polysciences). Two days after transfection, cells were stained with allophycocyanin-conjugated anti-FLAG (M2, Biolegend) and phycoerythrin-conjugated anti-HA monoclonal antibody (16B12, Biolegend) and analyzed by Attune NxT flow cytometer (Thermo Fisher Scientific).

### Protein expression and purification of trMincle

Bacterial expression and purification of the trMincle ectodomain was modified with the methods as described previously (Ishizuka et al. 2023). Briefly, the DNA fragment encoding Asn38–Pro180 of trMincle was cloned into a pGMT7 vector. The resulting plasmids were transformed into *E. coli* BL21 (DE3) competent cells, Champion21 (SMOBIO). Protein expression was induced by the addition of 1 mM isopropyl-β-D-thiogalactopyranoside (IPTG) and incubated at 18 °C overnight after induction. Cells were collected and disrupted with sonication. As trMincle ectodomain was expressed in inclusion bodies, insoluble fractions were collected and treated with lysozyme and DNase I. Then, the insoluble fraction was further washed with Triton wash buffer (50 mM Tris–HCl (pH 8.0), 0.1 M sodium chloride, 10 mM EDTA, and 0.5% (v/v) Triton X-100). The purified inclusion bodies were subsequently solubilized with denaturizing buffer (0.2 M Tris–HCl (pH 8.0), 6 M guanidine HCl, 10 mM EDTA, and 5 mM dithiothreitol). Seventy milligrams of the solubilized protein was rapidly diluted with 1 L of refolding buffer (0.1 M Tris–HCl (pH 8.0) and 1.0 M L-arginine, 5 M urea, 1 mM calcium chloride, 5 mM reduced glutathione, and 0.5 mM oxidized glutathione) at 4 °C. The refolded proteins were dialyzed against 10 mM Tris–HCl (pH 8.0) and 1 mM calcium chloride for 2 days. The dialyzed solution was filtered and applied onto HiTrap Q HP column (Cytiva) for anion exchange chromatography using A buffer (20 mM Tris–HCl (pH 8.0)) and B buffer (20 mM Tris–HCl (pH 8.0), 1 M sodium chloride). The trMincle ectodomain was collected and concentrated to up to 5.7 mg/mL by Vivaspin 500 (molecular weight cutoff 3 kDa, Sartorius). The purity of the protein was assessed by SDS-PAGE and Coomassie brilliant blue staining.

### Crystallization, data collection, and structure determination of trMincle

Crystallization trials were performed using the sitting drop vapor diffusion method. To obtain trMincle-glucose complex crystals, purified trMincle ectodomain (final 5.7 mg/mL) was mixed with D-glucose (final 100 mM) and incubated at 4 °C overnight prior to crystallization. Initial screening for crystallization conditions was searched using Index (Hampton Research). The best diffraction-quality crystals were grown under the conditions of 0.1 M Tris–HCl (pH 8.5), 0.2 M sodium chloride, and 25% (w/v) polyethylene glycol 3350. For X-ray diffraction experiments, the crystals were rapidly frozen in liquid nitrogen in the presence or absence of 20% (v/v) glycerol. X-ray diffraction datasets were collected at the beamlines BL-1A and BL-17A in Photon Factory, Tsukuba, Japan. Diffraction spots were integrated with the program XDS (Kabsch 2010) and scaled with the program AIMLESS (Evans and Murshudov 2013). Initial phase determination was performed by the molecular replacement method using the program MOLREP (Vagin and Teplyakov 2010). A hypothetical model of trMincle generated with AlphaFold3 (Abramson et al. 2024) was used as a search model. Model building was performed manually using the program COOT (Emsley et al. 2010). Refinement was initially conducted using REFMAC5 (Murshudov et al. 1997) and Phenix.refine (Liebschner et al. 2019) for the final model. The clear electron density corresponding to glycerol, not glucose, was observed in the sugar-binding site when a high concentration of glycerol was used as cryoprotectant. Therefore, this dataset was treated as a trMincle-glycerol complex. In contrast, the electron density corresponding to glucose was clearly observed in the absence of glycerol. We thus assigned this dataset as the trMincle-glucose complex. The stereochemical quality was assessed by MolProbity (Chen et al. 2010). Data collection and refinement statistics are summarized in Supplementary Table 1. All structural figures were generated using PyMOL (The PyMOL Molecular Graphics System, Version 2.0, Schrödinger, LLC). Structural superposition was performed with the program SUPERPOSE (Krissinel and Henrick 2004).

### Molecular dynamics simulation of trMincle

The trMincle CRD (Asn59–Pro180) (PDB ID: 9KS7) was used for the simulation. Glycerol was removed from the structure upon optimization of the structural data, prior to the simulation. Molecular dynamics simulations were performed using OpenMM 8 (Eastman et al. 2024). The AMBER14 force field (Maier et al. 2015) and the TIP3P-FB (Wang et al. 2014) were used for the force field and the water model, respectively. Long-range nonbounded interactions, interactions that include a repulsion term, a dispersion term, a Coulomb term, or van der Waals interactions, were computed in periodic boundary conditions. The Particle Mesh Ewald method (Darden et al. 1993) was used for computing long-range Coulomb interactions. The lengths of bonds involving a hydrogen atom are kept fixed, and water molecules are kept rigid during the simulation. All systems were kept at a temperature of 300 K and a pressure of 1.0 atmosphere. Equilibration length was held for 10,000 steps and simulation length was held for 1,000,000 steps (4 femto seconds per steps). Graphical representations of the molecular systems were also created with PyMOL.

### Phylogenetic analysis of huMincle homologues

To search for the huMincle homologues, BLAST search was performed in amphibians and reptiles using the huMincle protein sequence as a query. Amino acid sequences obtained from the top 10 hits from each BLAST search and amino acid sequence of trMincle, bovine Mincle, huMincle, and human Dectin-1 were aligned using MUSCLE (Edgar 2004). Based on the alignments, a phylogenetic tree was inferred by using the maximum likelihood method and the JTT matrix-based model (Jones et al. 1992). Initial tree(s) were obtained automatically by applying the Neighbor-Joining algorithm and BioNJ algorithms to a matrix of pairwise distances estimated using the JTT model. Next, the topology with superior log likelihood values was selected. Out of these initial trees, the tree with the highest log likelihood (− 2750.75) is shown in Fig. 4A. To model evolutionary rate differences among sites (5 categories (+ G, parameter = 2.4398)), a discrete Gamma distribution was used. According to analysis of the rate variation model involving 24 amino acid sequences, 7.27% of sites were evolutionarily invariable sites ([+ I], 7.27% sites). The final dataset contained 110 positions, and all positions containing gaps and missing data had been eliminated via the complete deletion option. These analyses were conducted in MEGA11 (Stecher et al. 2020); (Tamura et al. 2021).

## Results

### Mincle homologues in lower vertebrates

To find proteins homologous to Mincle in non-mammalian vertebrates, we searched for molecules with similar characteristics to mammalian Mincle in terms of amino acid sequence. Mincle in mammals shares several characteristics in their primary structures, namely the EPN (Glu-Pro-Asn) motif for recognition of glucose and mannose and a hydrophobic groove that binds acyl chains of glycolipids (Feinberg et al. 2013); (Furukawa et al. 2013).

**Fig. 1:**
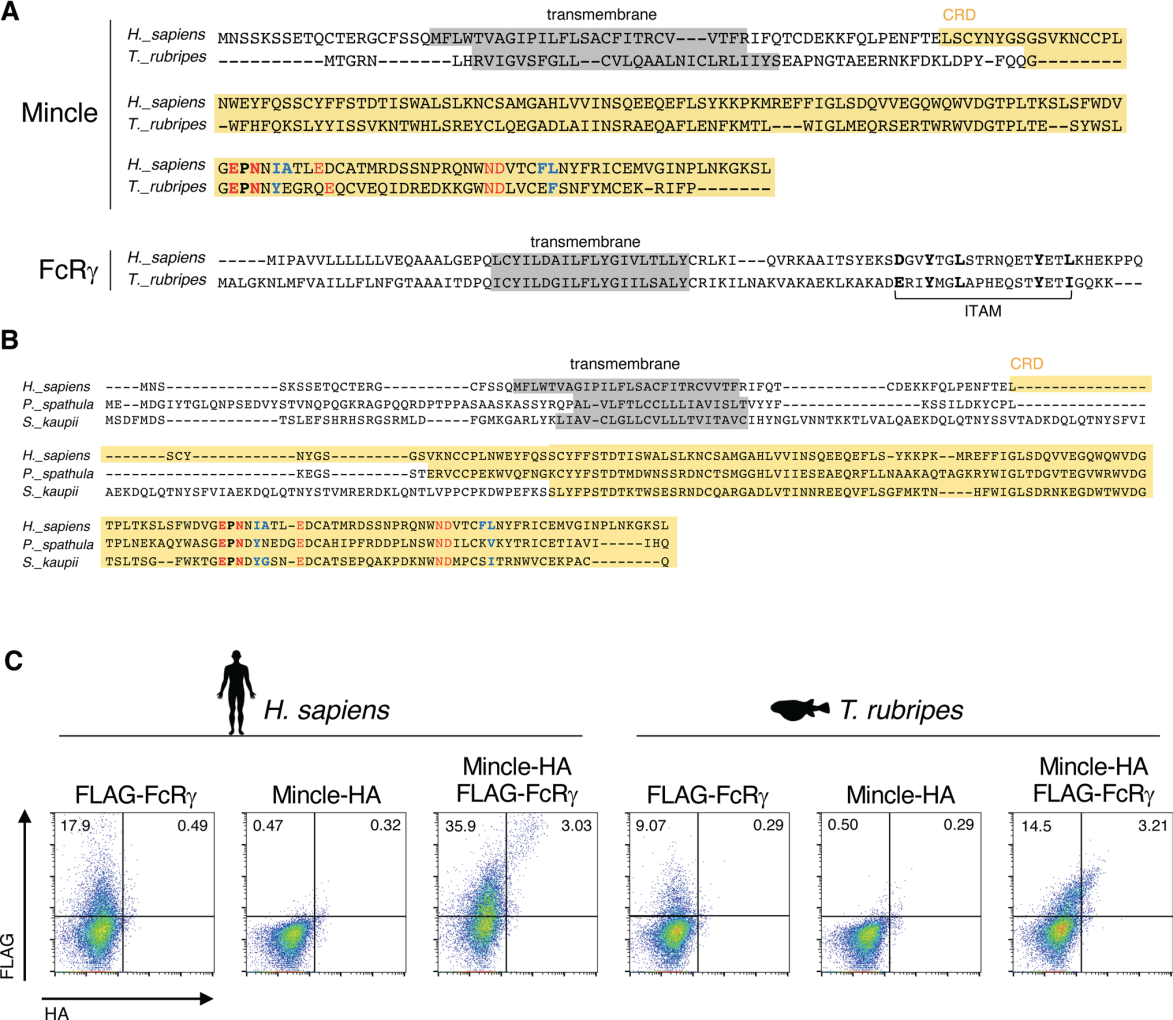
Mincle homologue in *Takifugu rubripes.* **A** Amino acid sequence alignment of full-length Mincle and FcRγ from human and *T. rubripes*. The transmembrane region and the CRD are shaded in gray and yellow, respectively. Amino acid residues that interact with a calcium ion are shown in red, hydrophobic groove-forming residues are shown in blue, and the characteristics of the mammalian Mincle are indicated in bold. The immunoreceptor tyrosine-based activation motif (ITAM) is labeled below the amino acid sequence. **B** Amino acid sequence alignment of full-length huMincle and Mincle homologues in *P. spathula* and *S. kaupii*. The transmembrane region and the CRD are shaded in gray and yellow, respectively. Amino acid residues that interact with a calcium ion are shown in red, hydrophobic groove-forming residues are shown in blue, and residues that are defined as the characteristics of the mammalian Mincle are indicated in bold. **C** Flow cytometry analysis showing the cell surface expression levels of HA-tagged Mincle and FLAG-tagged FcRγ on HEK293 transfectants. The species are indicated above. Data are representative of three independent experiments

We searched for human Mincle (huMincle) homologues in fish, one of the evolutionarily farthest vertebrates from mammals. In *Takifugu rubripes*, one of the first vertebrates whose whole genome was completely sequenced, a protein sequence bearing the EPN motif and a hydrophobic groove was found (XP_029706534.1) (Fig. 1A). Other bony fishes also had protein sequences that bear these Mincle characteristics, like *T. rubripes* (e.g., C-type lectin domain family 4 member E-like in *Polyodon spathula* (Mississippi paddlefish) and hypothetical protein SKAU_G00319680 from *Synaphobranchus kaupii* (Kaup’s arrowtooth eel)) (Fig. 1B). We further BLAST searched for Mincle homologues in sequences across vertebrates except for mammals. In the top 1000 hits, protein sequences from reptiles, amphibians, and bony fishes were included (Supplementary Table 2) and some possessed the amino acid sequence characteristics of mammalian Mincle. These results suggest that Mincle homologues possessing an EPN motif and hydrophobic groove might be present throughout jawed vertebrates.

As mammalian Mincle relies on FcRγ to signal, we further searched for FcRγ in *T. rubripes*. Indeed, there was a FcRγ homologue containing a conserved ITAM motif, consistent with the previous study (Guselnikov et al. 2003) (Fig. 1A). We then BLAST searched for human FcRγ (huFcRγ) in lower vertebrates, which revealed its sequence to be also well-conserved in reptiles, amphibian, and fishes, inferring the presence of FcRγ interacting CLRs, like Mincle (Supplementary Table 3). This suggests that in evolutionarily distant non-mammalian vertebrates, specifically fish, Mincle could interact with FcRγ to function, similar to mammalian Mincle.

As we found Mincle and FcRγ-like protein sequences in *T. rubripes*, we next set out to confirm the interaction of these proteins in vitro. Mammalian Mincle is not expressed on the cell surface without the interaction with FcRγ (Yamasaki et al. 2008). Thus, we ectopically expressed *T. rubripes* Mincle (trMincle) in the presence or absence of *T. rubripes* FcRγ (trFcRγ) in HEK293 cells and compared the surface expression of trMincle. Cell surface expression of trMincle was detected only in the presence of trFcRγ (Fig. 1C) suggesting that trMincle and trFcRγ behave similarly to mammalian Mincle and FcRγ in terms of surface expression at least in mammalian cells.

Altogether, trMincle shares similar characteristics with mammalian Mincle in its amino acid sequence and its FcRγ-dependent cell surface expression. This is despite the fact that trMincle is automatically annotated as a CD209 antigen-like protein A by the gene prediction method, Gnomon. However, the characteristics such as the short intracellular region without any signaling motifs, the conserved hydrophobic groove-forming amino acids, and the FcRγ-dependent cell surface expression of trMincle suggest that this protein is more likely a homologue of Mincle rather than CD209.

### Structural analysis of trMincle

**Fig. 2:**
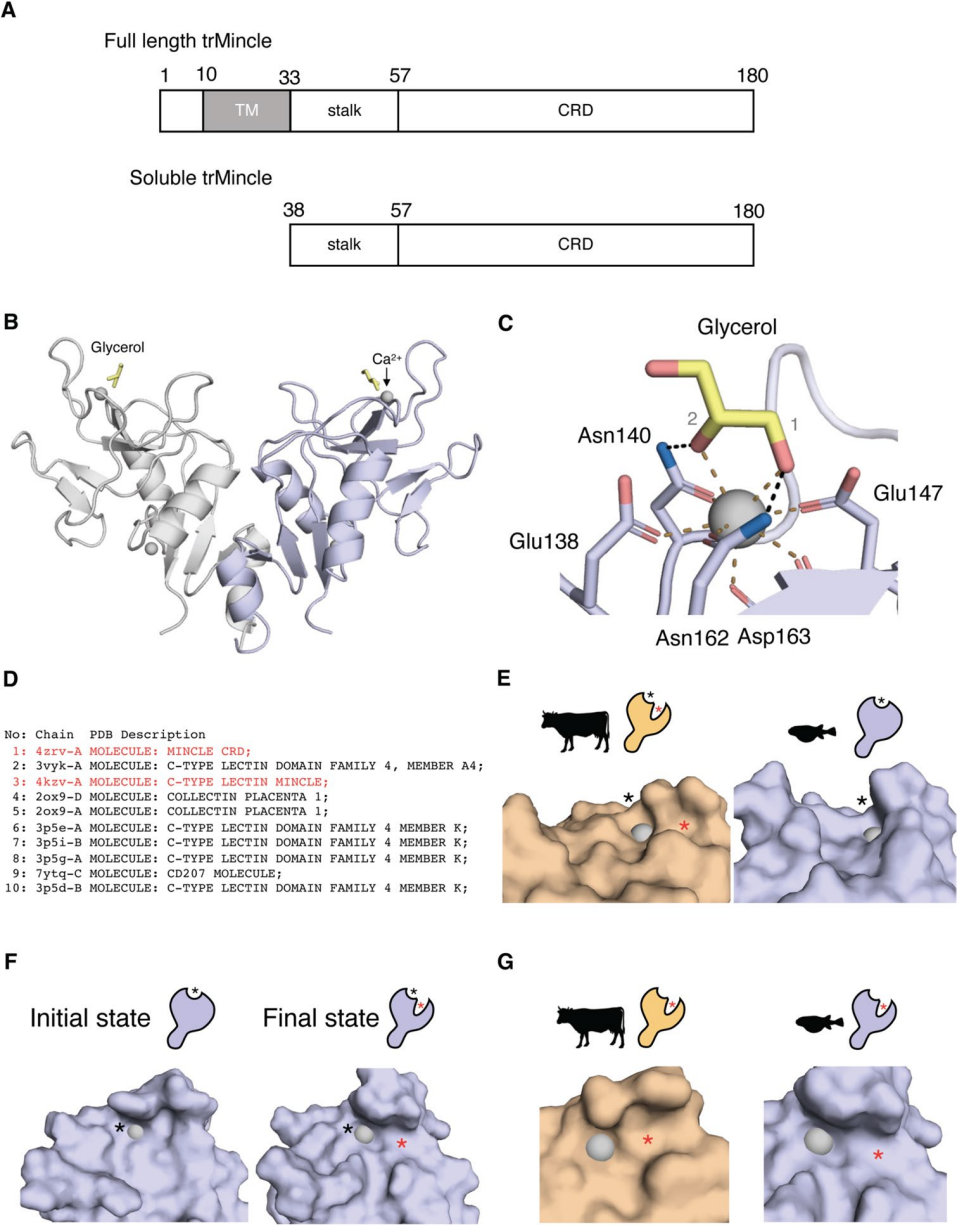
trMincle is structurally similar to boMincle. **A** Schematic representation of full-length trMincle (upper) and soluble trMincle (lower). TM, transmembrane helix; CRD, carbohydrate recognition domain.** B** Overall structure of the trMincle CRD homodimer in complex with glycerol (PDB ID: 9KS7). Protein, glycerol, and calcium ions are shown in ribbon, stick, and sphere models, respectively. **C** Close-up view of the glycerol binding site in trMincle. The hydroxyl groups 1 and 2 of the glycerol are labeled in gray. Coordination bonds are indicated by brown dotted lines, and hydrogen bonds are indicated by black dotted lines.** D** List of the top ten PDB structures that are structurally similar to trMincle obtained from a full PDB search of Dali server. PDB IDs and chain names are indicated in the chain column. Descriptions of each structure are indicated in the PDB Description column. Mincle in the list is highlighted in red. **E** Surface representation of overall ligand binding region of boMincle (PDB ID: 4KZV) (left panel) and trMincle (PDB ID: 9KS7) (right panel). Schematic drawings of the receptor showing representative grooves are given above the panel. Asterisks indicate the corresponding grooves in the structure. **F** Representative state of trMincle ligand binding region inferred from molecular dynamics simulation. The left panel is the initial state, while the right panel shows the final state of the simulation. These images are depicted from the same view angle. **G** Comparison of the position of the hydrophobic groove on boMincle (left panel) and the putative hydrophobic groove on trMincle in the final state (right panel). Calcium ion is represented as a gray sphere

Next, to compare the 3D structural similarity of trMincle with mammalian Mincle, a structural analysis of trMincle was performed. As trMincle was predicted to be a type II transmembrane protein (Supplementary Fig. 1A, B), we constructed the extracellular domain of trMincle as a soluble recombinant protein (Fig. 2A) followed by column-based purification. The crystal structure of trMincle was determined at 1.7 Å resolution (Fig. 2B). A calcium ion was coordinated with Glu-Pro-Asn (EPN) and Trp-Asn-Asp (WND), two well-conserved motifs, suggesting the typical sugar-binding pocket observed in other mammalian C-type lectins was also present in trMincle. Furthermore, one glycerol molecule in the media interacted with the EPN motif within the hydrophilic pocket (Fig. 2B), and hydroxyl groups at C1 and 2 of glycerol formed coordination bonds with calcium ion (Fig. 2C), which is similar to the reported sugar-binding mode of mammalian Mincle (Feinberg et al. 2013).

Using this first structure of Mincle homologue in non-mammalian species, we conversely searched for homologues based on structural similarity. As expected, a Dali server search of all deposited structures in the Protein Data Bank (PDB) revealed that mammalian Mincle, such as the bovine Mincle (boMincle) CRD (PDB ID: 4KZV), was the most structurally similar protein to the trMincle CRD (Fig. 2D). Indeed, overall, both fish and mammalian Mincle possessed hydrophilic pockets and adjacent grooves (Feinberg et al. 2013); (Furukawa et al. 2013), although the amino acid residues were not identical (Figs. 1B and 2E, Supplementary video 1, 2). Moreover, the molecular dynamics simulation suggested that the trMincle CRD can create a typical groove similar to a mammalian Mincle (Fig. 2F, G, Supplementary video 3).

### trMincle possesses narrow sugar-binding pocket

**Fig. 3:**
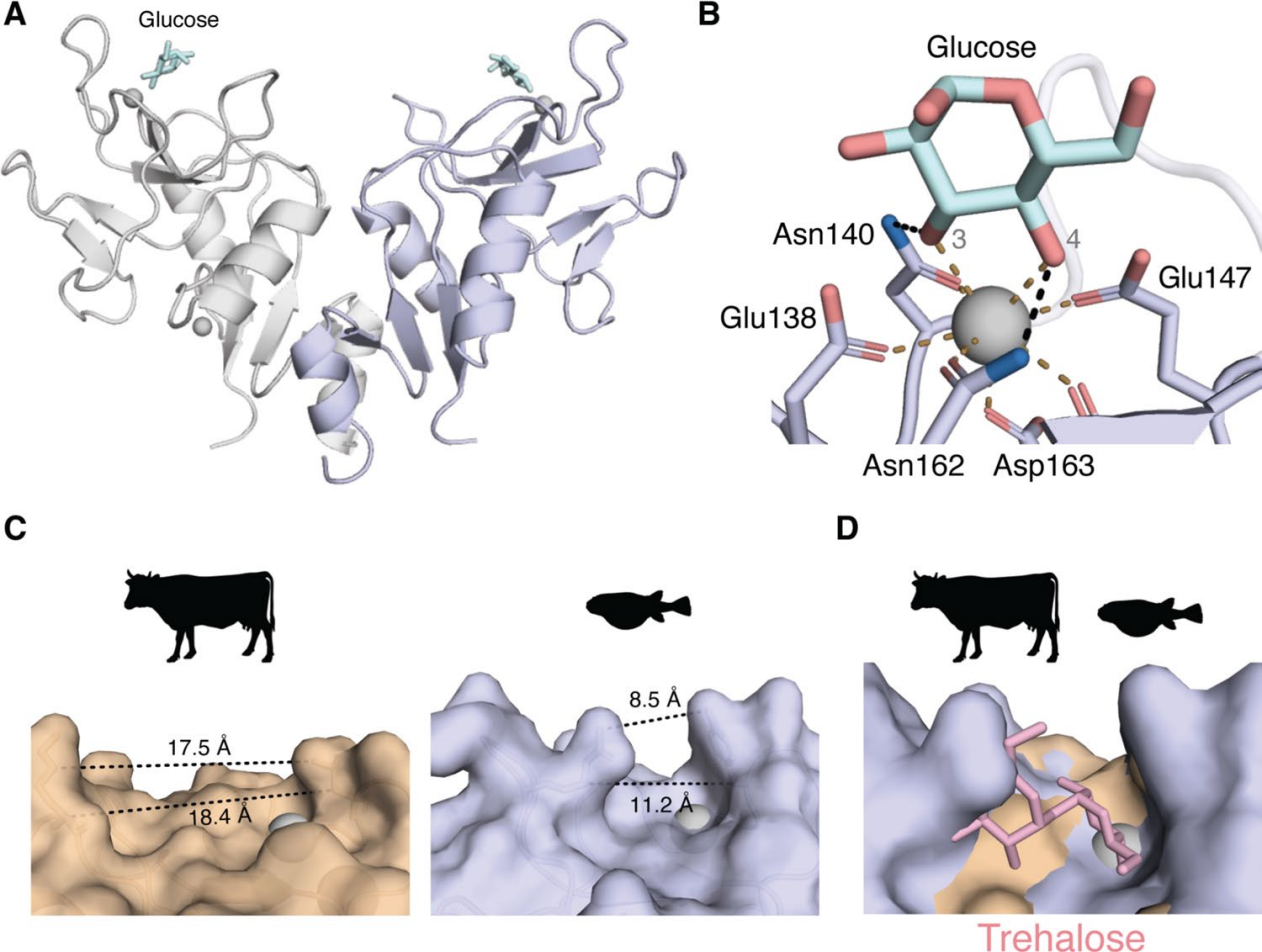
Structural differences in the sugar-binding pocket. **A** Overall structure of the trMincle CRD homodimer in complex with glucose (PDB ID: 9KPL). Proteins, glucose, and calcium ions are shown in ribbon, stick, and sphere models, respectively. **B** Close-up view of the glucose binding site in trMincle. The hydroxyl groups 3 and 4 of the glucose molecule are labeled in gray. Coordination bonds are indicated by brown dotted lines, and hydrogen bonds are indicated by black dotted lines.** C** Distances of amino acid residues shaping the sugar-binding pocket. The measured distances are indicated near the dotted lines. **D** Overlayed structure of trMincle and boMincle-trehalose complex. Structures were superposed according to the position of the calcium ions in their sugar-binding pockets. Surface of the boMincle and trMincle is shown in orange and blue, respectively. The calcium ion is shown as a gray sphere

Mammalian Mincle binds calcium ion via an EPN motif which is responsible for its sugar-binding ability. As the structural analysis suggested that trMincle possesses a sugar-binding pocket (Fig. 2B), we set out to investigate the sugar recognition mechanism using monosaccharide glucose as a candidate for the model ligand. Indeed, the crystal structure of the trMincle CRD-glucose complex was determined at 1.8 Å resolution (Fig. 3A). As suggested by the glycerol complex, one glucose molecule interacted with the calcium ion as well as the surrounding side chains (Fig. 3B). Thus, the sugar-binding mode of trMincle was similar to the other CLRs bearing EPN motifs (Drickamer and Taylor 2015) (Fig. 3B). In contrast, the sugar-binding pocket of trMincle was narrower than that of mammalian Mincle, such as boMincle (PDB ID: 4ZRW) and huMincle (PDB ID: 3WH2) (Fig. 3C), suggesting that trMincle preferentially binds monosaccharide, not disaccharide. This is in marked contrast to mammalian Mincle, which has the capacity to recognize trehalose disaccharide (Ishikawa et al. 2009); (Feinberg et al. 2013).

Indeed, computational superposition of the boMincle-trehalose complex onto the trMincle-glucose complex demonstrated that trehalose causes a heavy steric clash with trMincle, suggesting that trehalose disaccharide cannot bind to trMincle due to the narrow pocket (Fig. 3D). It evokes the possibility that trMincle cannot recognize disaccharide-bearing glycolipids, which are typical of exogenous origin, such as mycobacterial glycolipids (e.g., TDM or trehalose monomycolate (TMM)), but instead may only bind monosaccharide-bearing glycolipids, which are often derived from self, like β-GlcCer (Nagata et al. 2017). While mammalian Mincle recognizes both exogenous diglycosylated lipids and endogenous monoglycosylated lipids, our results imply that trMincle may function as a receptor that selectively binds ligands such as monosaccharide-bearing glycolipids.

### Comparing the structural models of Mincle homologues across reptiles and amphibians

**Fig. 4:**
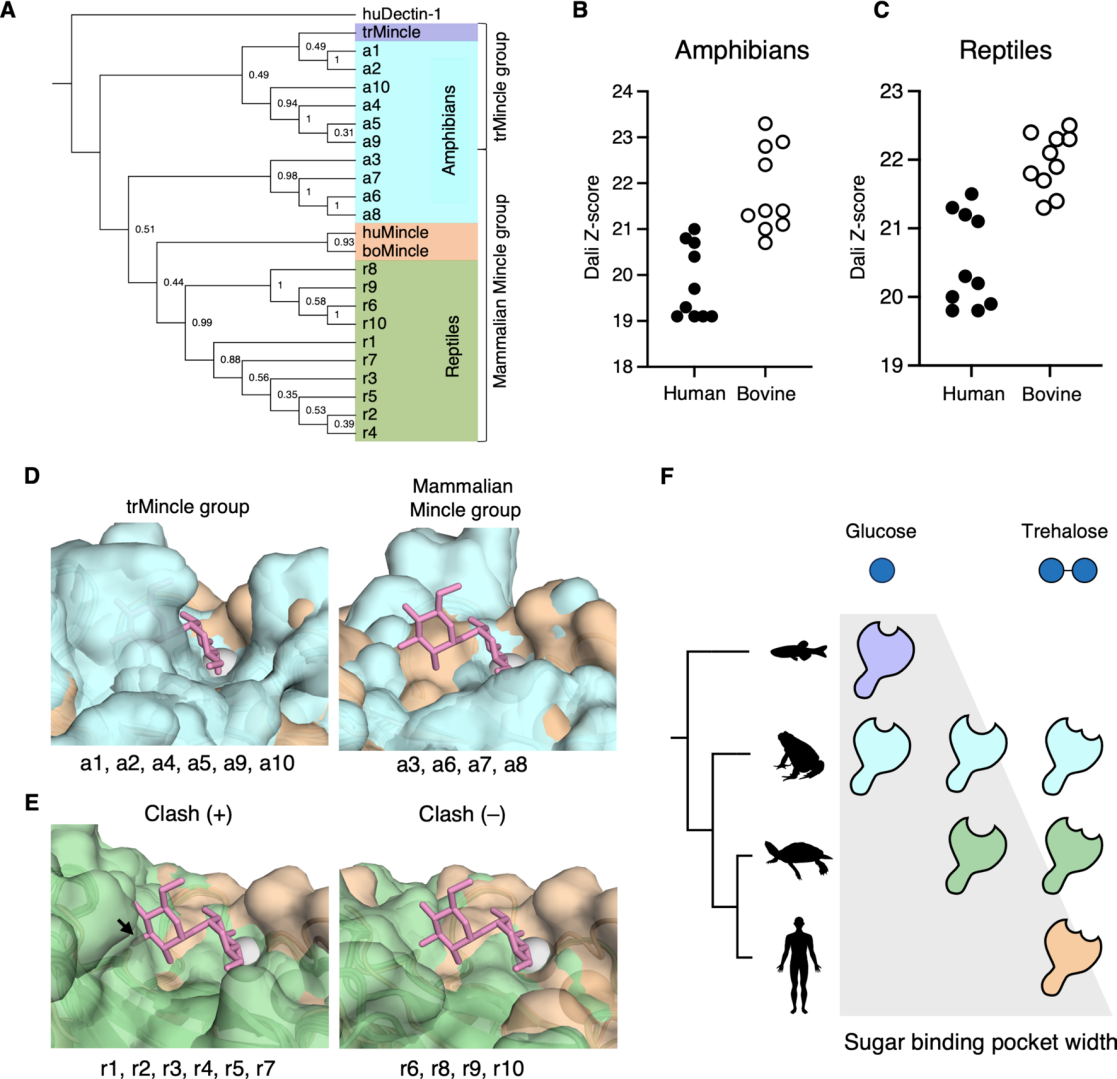
Evolution of ligand binding region of Mincle CRD. **A** Maximum likelihood tree for amino acid sequences inferred from the BLAST top 10 hits for searching Mincle homologues in amphibians and reptiles. The numbers beside each node indicate bootstrap values calculated with 1000 replications. Amphibian-derived molecules and reptile-derived molecules are highlighted in cyan and green, respectively. Molecules in the same clade with trMincle are indicated as the trMincle group, and those in the same clade with huMincle and boMincle are indicated as the mammalian Mincle group. Human Dectin-1 (huDecin-1) was used as an outgroup. Dali Z-score distributions of** B** amphibian Mincle homologues and **C** reptilian Mincle homologues in Dali search. Dali Z-scores were calculated by comparing the structures of the AlphaFold3-predicted CRD structure of Mincle homologues and the crystal structure of huMincle or boMincle.** D** Superposition of boMincle (PDB ID: 4KZV) (brown) and amphibian Mincle homologues (cyan). The structures were superposed according to the positions of calcium ions in their putative sugar-binding pockets. Structures in trMincle groups and mammalian Mincle groups are shown in the left and right panels, respectively. Individual structures viewed from the same angle are shown in Supplementary Fig. 2C. Trehalose bound in the sugar-binding pocket of boMincle is shown in a stick model. Calcium ion is shown in a sphere model.** E** Superposition of boMincle (PDB ID: 4KZV) (brown) and reptilian Mincle homologues (green). The structures are superposed according to the positions of calcium ions in their putative sugar-binding pockets. Structures that have steric clashes with trehalose (clash (+)) and those without steric clash (clash (–)) are shown in the left and right panels, respectively. The arrow in the clash (+) panel indicates the location of the clash observed in the structure. Structures of the sugar-binding pocket in individual reptilian protein viewed from the same angle are shown in Supplementary Fig. 2D. Trehalose and calcium ions are shown in a stick model and in a sphere model, respectively. **F** Scheme that shows the widening of the sugar-binding pocket and the broadened capacity of expected ligands during evolution

As mentioned, proteins with characteristics of huMincle are conserved among several species. To gain an insight into how Mincle evolved between fish and mammals, we first searched for homologous proteins in amphibians and reptiles respectively using protein BLAST (Supplementary Table 4, 5). Phylogenetic analysis of the top 10 hits of amphibians and reptiles was conducted to check the amino acid sequence similarity to huMincle and boMincle (Fig. 4A). All reptile-derived molecules and four out of ten amphibian molecules were classified in the same clade with mammalian Mincle. As the other six amphibian homologues were grouped into the same clade as trMincle, structure predictions with AlphaFold3 were performed. Dali server was used to analyze the overall structural similarity between predicted structures and the huMincle and boMincle crystal structures (PDB ID: 3WH2, 4KZV). Dali Z-score, a score that provides information on structural similarity, was higher for boMincle than that of huMincle, in both species (Fig. 4B, C, Supplementary Fig. 2A, B). Amphibian molecules were divided into two clusters based on their Dali Z-score (Fig. 4B). The higher Dali Z-scored proteins were in the same clade as mammalian Mincle, while the other lower Dali Z-scored proteins were in the same clade with trMincle (Fig. 4A, Supplementary Fig. 2A).

Next, to compare the sugar-binding pocket, we superposed trehalose-bound boMincle CRD structures (PDB ID: 4KZV) onto predicted structures of amphibian and reptile homologues, by aligning the sugar-binding pockets’ calcium ions (Fig. 4D, E). Amphibian Mincle in the same clade as the mammalian Mincle had broad sugar-binding pockets, big enough to bind disaccharides (Fig. 4D, Supplementary Fig. 2C). In contrast, amphibian Mincle in the same clade with trMincle had narrower binding pockets, similar to trMincle (Fig. 4D, Supplementary Fig. 2C). Reptile Mincle had, overall, broader sugar-binding pockets than trMincle; however, some still had steric clashes with a trehalose (Fig. 4E, Supplementary Fig. 2D). In sharp contrast to mammalian Mincle that possessed a wide sugar-binding pocket, most of the fish Mincle were predicted to possess narrower pockets (Supplementary Fig. 2 E, F). Proteins with or without steric clashes were further classified as two distinct groups according to the phylogenetic analysis (Fig. 4A, E). These data suggest the widening of the sugar-binding pocket during evolution.

## Discussion

In this study, we found that Mincle homologues are present among bony fishes, amphibians, and reptiles. Structural comparisons including experimentally determined and predicted models suggested that Mincle originally arose to recognize monosaccharide-bearing glycolipids, presumably self-derived glycolipids, and eventually acquired the capability to recognize broader ligands such as pathogen-derived disaccharide-bearing glycolipids (Fig. 4F).

It would also be intriguing to consider the possibility of convergent evolution; however, trMincle shared multiple characteristics and motifs with mammalian Mincle, making this possibility less likely.

The crystal structure of trMincle suggests the formation of a stable homodimer. Homodimerization has been reported recently for huMincle (Liu et al. 2024), which probably increases valency to compensate for CLR’s relatively low ligand binding affinity (Guenther et al. 2022). Heterodimer formation with macrophage C-type lectin (MCL), another TDM-recognizing CLR, has also been reported, as was Mincle and MCL functioning cooperatively (Lobato-Pascual et al. 2013); (Miyake et al. 2015). Although no MCL-equivalent molecule has been identified in fish, it is possible that receptors forming heterodimers, similar to MCL, might exist in fish. Alternatively, Mincle may have originally functioned as a homodimer and acquired cooperative molecules during evolution, as CLRs may have diversified through gene duplication (Sattler et al. 2012); (Drickamer and Taylor 2015); (Miyake et al. 2015).

The narrow sugar-binding pocket of trMincle suggests specialized recognition for monosaccharides. This makes it difficult to recognize well-known mammalian Mincle ligands such as mycobacterial glycolipid TDM. On the other hand, infections caused by the *Mycobacterium* species have been widely reported in fish (Decostere et al. 2004). These infections can be lethal in fish (Sar et al. 2004), suggesting the need for mechanisms to detect mycobacterial infections. Indeed, immune responses against formalin-killed mycobacteria and mycobacteria-derived cell wall glycolipids have been reported in fish (Kato et al. 2010); (Matsumoto et al. 2018). Although trMincle may not have the ability to recognize typical mycobacterial glycolipids like mammals, it may detect fatal infection through the alteration of endogenous metabolites, such as hexosylceramides (Bedard et al. 2023).

Although the structure of the sugar-binding pocket has changed drastically from fish to mammals, we could not address the possible driving force of the structural changes. Changes in microorganisms exposed to environmental selective pressures might have affected ligand specificity. The rapid evolution of CLRs has been reported in separate mammalian lineages: primates, rodents, and bats (Hilbert et al. 2023). These different lineages showed different patterns of amino acid selection suggesting the involvement of distinct populations of microbial species in shaping the evolution of these receptors. They proposed this extensive evolution to be a result of the host–pathogen arms race. Adaptation to terrestrial environments possibly increased the chance to be exposed to new kinds of microorganisms that may function as the driver for such evolution.

Although putative CLRs similar to Mincle are found across fish species, EPN motifs were not faithfully conserved in fish species, such as *Danio rerio*, *Oryzias latipes*, *Lates calcarifer*, and *Lutjanus peru*. Intriguingly, Mincle-like protein in *Lates calcarifer* and *Lutjanus peru* had galactose-type QPD and unknown-type EPD motif instead of glucose/mannose-type EPN motifs in the corresponding position (Zoccola et al. 2017); (Guluarte et al. 2022), implying the different ligand specificity in these species. Even between mouse and human, there are different numbers of genes in the Dectin-2 cluster, a gene cluster that includes the Mincle gene (Malamud and Brown 2024), suggesting active species-specific gene duplication and diversification took place. In contrast, FcRγ is highly conserved among species (Yoder et al. 2007). Despite the diversity, interaction with FcRγ is conserved among Dectin-2 cluster genes in mammals. In contrast, the number of genes in the Dectin-1 cluster, a CLR gene cluster that includes Dectin-1, has not changed between mouse and human (Malamud and Brown 2024). As CLRs in the Dectin-1 cluster do not need association with adaptor proteins, like FcRγ, separation of a signaling unit might allow drastic mutation during evolution which may drive the acquisition of diverse gene clusters.

Phylogenetic analysis grouped Mincle homologues in amphibians into two groups, the trMincle group, which has trMincle-like sugar-binding pockets, and the mammalian Mincle group, which has mammalian-like broad sugar-binding pockets. The trMincle group proteins are derived from species that branched out early, such as *Hyperolius riggenbachi* and *Xenopus tropicalis *(Portik et al. 2023). In contrast, the mammalian Mincle group proteins derived from species that branched out relatively recently, such as *Spea bombifrons* and *Bufo gargarizans *(Portik et al. 2023).

Although further analysis on other amphibians is needed, this supports our speculation that the sugar-binding pocket of Mincle has widened during the evolution to adapt to additional ligands.

## Supplementary Information

Below is the link to the electronic supplementary material.

Supplementary file1 (DOCX 7.16 MB)

Supplementary file2 (XLSX 22.3 KB)

Supplementary file3 (MP4 23.7 MB)

Supplementary file4 (MP4 24.1 MB)

Supplementary file5 (MP4 20.8 MB)

## Data Availability

Structure factors and the coordinates have been deposited in the Protein Data Bank database under the accession codes 9KS7 (trMincle-glycerol complex) and 9KPL (trMincleglucose complex). Other data are available upon request.
