# Supplementary material for: Phylogenetic and structural insights into the origin of C-type lectin Mincle in vertebrates
Source: Immunogenetics. 2025 Mar 22;77(1):18. doi: 10.1007/s00251-025-01375-x (PMC11929736; doi:10.1007/s00251-025-01375-x)
Supplement: Supplementary file 1 — Supplementary file1 (DOCX 7.16 MB) [file 251_2025_1375_MOESM1_ESM.docx]

**Supplementary Figures**

*Immunogenetics*

**Phylogenetic and structural insights into the origin of C-type lectin Mincle in vertebrates**

Taiki Ito^1,2^, Carla Guenther^2^, Eri Ishikawa^1,2^, Takae Yabuki^1,3^, Masamichi Nagae^1,2^, *Yoichiro Nakatani^4^ and *Sho Yamasaki^1,2,3,5^

^1^Department of Molecular Immunology, Research Institute for Microbial Diseases, Osaka University, Suita, Osaka, Japan; ^2^Laboratory of Molecular Immunology, Immunology Frontier Research Center (IFReC), Osaka University, Suita, Osaka, Japan; ^3^Center for Advanced Modalities and Drug Delivery Systems (CAMaD), Osaka University, Suita, Osaka, Japan; ^4^Laboratory of Medical and Evolutionary Genomics, Department of Biological Informatics, Bioinformatics Center, Research Institute for Microbial Diseases, Osaka University, Suita, Osaka, Japan; ^5^Center for Infectious Disease Education and Research (CiDER), Osaka University, Suita, Osaka, Japan

*To whom correspondence should be addressed:

Yoichiro Nakatani: nakatani@biken.osaka-u.ac.jp

Sho Yamasaki: yamasaki@biken.osaka-u.ac.jp

**

**

**Supplementary Fig. 1. trMincle is predicted to be a type II transmembrane protein.**

**(A)** Transmembrane prediction of trMincle using CCTOP web server (Dobson, et al. 2015). The topology is indicated as follows: the cytosol (red), transmembrane (gray) and extracellular side (blue). (**B)** Transmembrane prediction results in other methods (Krogh, et al. 2001; Tusnady and Simon 2001; Viklund and Elofsson 2004; Bernsel, et al. 2008; Viklund and Elofsson 2008; Nugent and Jones 2012). Names of the methods used are indicated on the left side. The meaning of colors is same as in (**A)**.


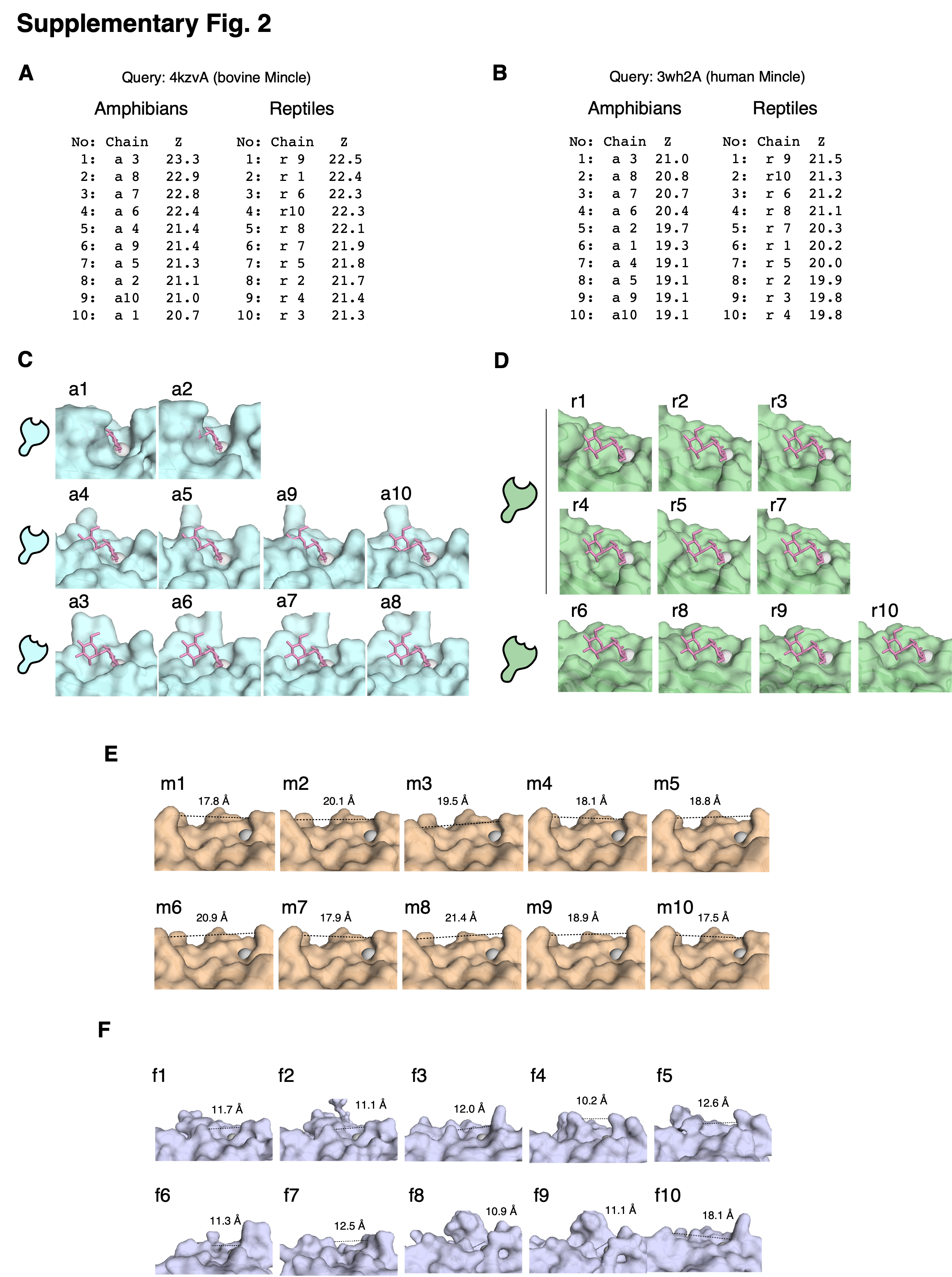


**Supplementary Fig. 2. Z-scores and sugar binding pocket of each Mincle homologue.**

**(A,B)** Dali Z-scores calculated from the structural comparison with the boMincle **(A)** and huMincle **(B)**. Results for amphibians and reptiles are placed as indicated. Numbers in the No. column are the ranks in this analysis. Numbers in the Chain column corresponds to the BLAST ranks, “a” stands for amphibian and “r” stands for reptile. Numbers in the Z-column shows the actual Dali Z-scores. **(C,D)** Individual structures of putative sugar binding pocket shown in Fig. 4D and E. The positions of trehalose are deduced from the superposition of boMincle-trehalose complex as shown in Fig. 4D and E. Schematic in the left of the each row indicates the broadness of the sugar binding pockets. The trehalose and calcium ion are shown in stick and sphere model, respectively. Structures are depicted from the same angle as the corresponding figures in Fig. 4. **(E,F)** Putative sugar binding pockets of AlphaFold predicted structures of top 10 BLAST hits of (**E**) mammals (not including human) and (**F**) fish. Full length huMincle amino acid sequence was used as query for the BLAST. The measured distances of amino acid residues forming sugar binding pocket are indicated near the dotted lines. The results of the BLAST are presented in the Supplementary table 6 (mammals) and 7 (fish).

**References**

Bernsel A, Viklund H, Falk J, Lindahl E, von Heijne G, Elofsson A. 2008. Prediction of membrane-protein topology from first principles. Proceedings of the National Academy of Sciences of the United States of America 105:7177-7181. https://doi.org/10.1073/pnas.0711151105

Dobson L, Remenyi I, Tusnady GE. 2015. CCTOP: a Consensus Constrained TOPology prediction web server. Nucleic Acids Res 43:W408-412. https://doi.org/10.1093/nar/gkv451

Krogh A, Larsson B, von Heijne G, Sonnhammer EL. 2001. Predicting transmembrane protein topology with a hidden Markov model: application to complete genomes. J Mol Biol 305:567-580. https://doi.org/10.1006/jmbi.2000.4315

Nugent T, Jones DT. 2012. Detecting pore-lining regions in transmembrane protein sequences. BMC Bioinformatics 13:169. https://doi.org/10.1186/1471-2105-13-169

Tusnady GE, Simon I. 2001. The HMMTOP transmembrane topology prediction server. Bioinformatics 17:849-850. https://doi.org/10.1093/bioinformatics/17.9.849

Viklund H, Elofsson A. 2004. Best alpha-helical transmembrane protein topology predictions are achieved using hidden Markov models and evolutionary information. Protein Sci 13:1908-1917. https://doi.org/10.1110/ps.04625404

Viklund H, Elofsson A. 2008. OCTOPUS: improving topology prediction by two-track ANN-based preference scores and an extended topological grammar. Bioinformatics 24:1662-1668. https://doi.org/10.1093/bioinformatics/btn221
